# An overview on inactivated and live‐attenuated SARS‐CoV‐2 vaccines

**DOI:** 10.1002/jcla.24418

**Published:** 2022-04-14

**Authors:** Saeed Khoshnood, Maniya Arshadi, Sousan Akrami, Maryam Koupaei, Hossein Ghahramanpour, Aref Shariati, Nourkhoda Sadeghifard, Mohsen Heidary

**Affiliations:** ^1^ Clinical Microbiology Research Center Ilam University of Medical Sciences Ilam Iran; ^2^ Department of Microbiology School of Medicine Ahvaz Jundishapur University of Medical Sciences Ahvaz Iran; ^3^ Infectious and Tropical Diseases Research Center Health Research Institute Ahvaz Jundishapur University of Medical Sciences Ahvaz Iran; ^4^ Student Research Committee Ahvaz Jundishapur University of Medical Sciences Ahvaz Iran; ^5^ 48462 Department of Microbiology and Immunology School of Medicine Kashan University of Medical Sciences Kashan Iran; ^6^ Department of Bacteriology Faculty of Medical Sciences Tarbiat Modares University Tehran Iran; ^7^ Molecular and Medicine Research Center Khomein University of Medical Sciences Khomein Iran; ^8^ 56941 Department of Laboratory Sciences School of Paramedical Sciences Sabzevar University of Medical Sciences Sabzevar Iran; ^9^ 56941 Cellular and Molecular Research Center Sabzevar University of Medical Sciences Sabzevar Iran

**Keywords:** attenuated vaccine, COVID‐19, inactivated vaccine, review, SARS‐CoV‐2

## Abstract

After about 2 years since severe acute respiratory syndrome coronavirus 2 (SARS‑CoV‑2), first infections were detected in Wuhan city of China in December 2019, which was followed by a worldwide pandemic with a record of 5.41 million deaths. Due to urgent need for the development of a safe and effective vaccine for coronavirus disease 2019 (COVID‐19), attempts for producing efficient vaccines are inexhaustibly continuing. According to a report by the World Health Organization (WHO) on COVID‐19 vaccine tracker and landscape, there are 149 vaccine candidates all over the world. Inactivated SARS‐CoV‐2 vaccines as a conventional vaccine platform consist of whole virus particles grown in cell culture and inactivated by chemicals. Because of benefits such as antigenic similarity to real virion inducing humoral and cellular immune responses and ease for transport and storage, these vaccines, including the vaccines produced by Bharat Biotech, Sinopharm, and Sinovac, are in use at large scales. In this study, we have a review on inactivated SARS‐CoV‐2 vaccines that are passing their phase 3 and 4 clinical trials, population which was included in the trials, vaccine producers, the efficiency, adverse effects, and components of vaccines, and other vaccine features.

## Graphical abstract

SARS‐CoV‐2 virus enters the body and replicates inside the cells. COVID‐19 is ingested by an antigen presenting cells like dendritic cells. Afterward, the antigen is recognized by Th cells that recruit other immune cells for infection control. B cells produce specific antibody against COVID‐19 and cytotoxic T cells destroy the cell infected by virus. Finally, some B and T cells remain in the body for immunological memory.

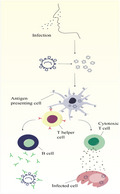

## 1 INTRODUCTION

**FIGURE 1:**
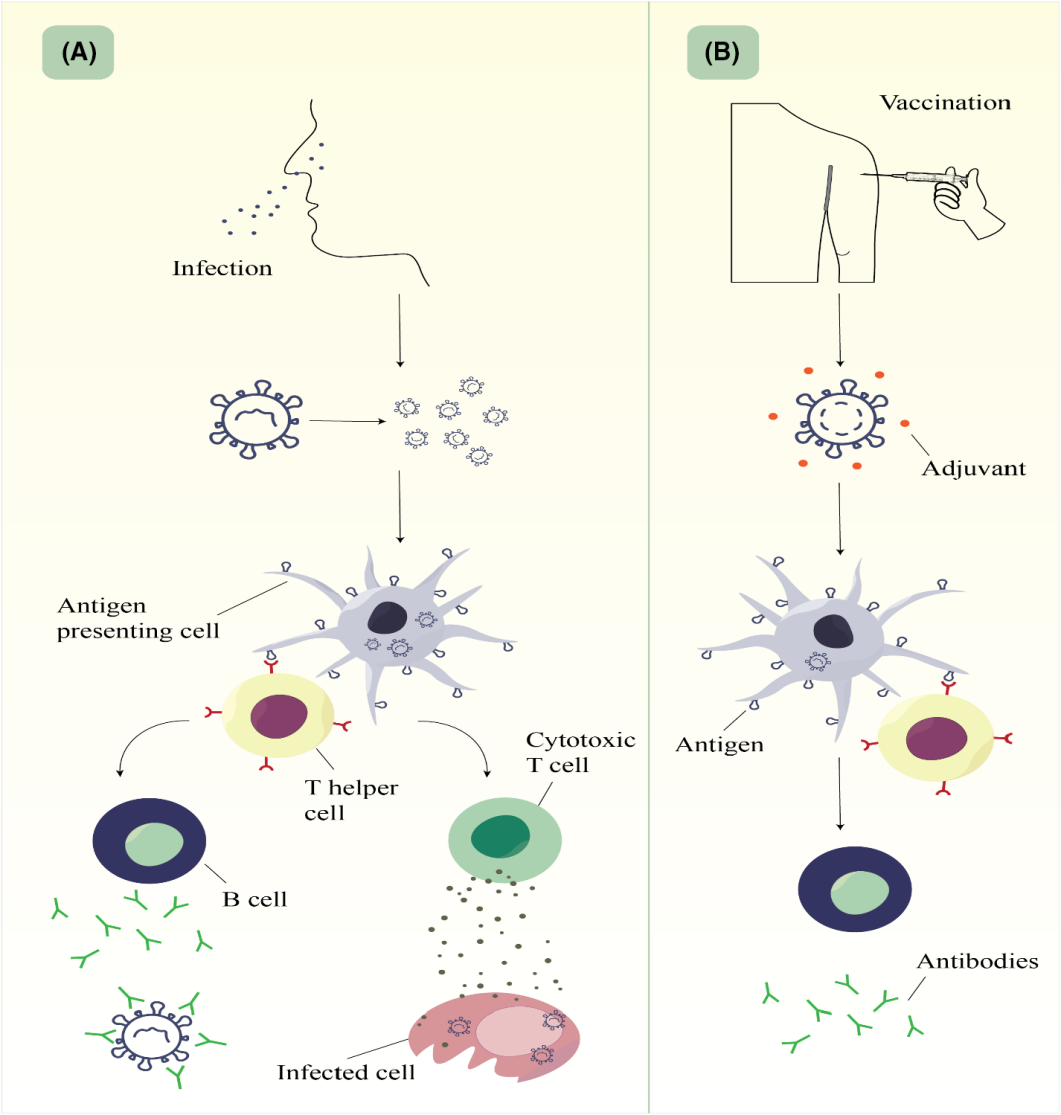
(A) Cellular and humoral immune responses in COVID‐19. This virus enters the body and replicates inside the cells. COVID‐19 is ingested by an antigen presenting cells like dendritic cells. Afterward, the antigen is recognized by Th cells that recruit other immune cells for infection control. B cells produce specific antibody against COVID‐19 and cytotoxic T cells destroy the cell infected by virus. Finally, some B and T cells remain in the body for immunological memory. (B) Mechanism of action of the inactivated COVID‐19 vaccines. Inactivated virus cannot replicate inside the body; therefore, higher doses are needed. Adjuvant could strength the immune responses. Noteworthy, inactivated vaccine generally induce antibody‐mediated immunity

The world community has been battling a global epidemic for about 2 years. Coronavirus disease 2019 (COVID‐19) is caused by the severe acute respiratory syndrome corona virus 2 (SARS‐CoV‐2).^1^ Recent studies have reported cellular and humoral immune responses to COVID‐19 in infected patients (Figure 1A,B). Due to the high‐rate transmission of the Middle East respiratory coronavirus compared with SARS‐CoV‐2, the need for an immediate vaccine design for this virus is exceedingly felt.^2^

Vaccines are used for the prevention and treatment of COVID‐19.^3^ DNA‐based vaccines, RNA‐based vaccines, recombinant subunit vaccines, adenovirus‐based vectors, and inactivated viruses are various types of SARS‐CoV‐2 vaccines that have lately been developed.^4^ In many countries of the world, vaccination has been or is being carried out on a large scale, especially among frontline workers.^5^ According to a document prepared by the US Food and Drug Administration, COVID‐19 vaccines must have key attributes, for example, clinical data, toxicity, and description of immune responses in the animal model, to be licensed.^6^

Inactivated vaccines are produced through the growth of SARS‐CoV‐2 in the cell culture and subsequent inactivation of the virus.^7^ There are several methods of inactivation, such as the use of formaldehyde, glutaraldehyde, ultraviolet, and gamma rays.^8^ Therefore, a biosafety level 3 is required to produce inactivated vaccines. Different countries, including China, Kazakhstan, and India, have developed this type of vaccine. Inactivated vaccines are given intramuscularly. Immune responses are induced against the spike proteins, matrix, envelope, and nucleoprotein.^7^ The level of antibodies decreases over time, indicating the need for a long‐term study of the protective effect of inactivated vaccines.^9^ Ease of use is one of the benefits of using inactivated vaccines. So far, SARS‐CoV‐2 adjuvants have been evaluated in humans to be used for the improvement of immunogenicity.^10^ Another merit of inactivated vaccines is their high speed of development, which makes them a viable option for developing COVID‐19 vaccines.^2^ Moreover, inactivated vaccines can be stored at 2–8°C, making them suitable for countries with limited cold storage capacity.^11^ However, there are disadvantages such as the need for high levels of contagious virus.^10^ Virus antigens and epitopes may be destroyed during the inactivation process, leading to a weakened immune response.^8^ Inactivated vaccines have hitherto been developed for two viral diseases, influenza virus and poliovirus.^12^ As of June 21, 2021, 1049 doses of SARS‐CoV‐2 inactivated vaccine had been vaccinated in China.^13^ Due to genetic changes in the SARS‐CoV‐2 genome, the emergence of new strains of the virus is often observed. The effect of vaccines on these new strains has not yet been determined.^14^ With this in mind, this study was undertaken to evaluate the efficacy and immunogenicity of various inactivated vaccines produced against SARS‐CoV‐2.

## 2 COVI‐VAC VACCINE

COVI‐VAC is a single‐dose intranasal live‐attenuated vaccine against SARS‐CoV‐2 produced by the United Kingdom (UK)'s Codagenix and Serum Institute of India. Codagenix has introduced 283 silent mutations into the gene encoding the viral spike protein. As a live‐attenuated vaccine, COVI‐VAC presumably produces immunity against all SARS‐CoV‐2 proteins, not just the spike surface protein, protecting a range of SARS‐CoV2 strains. Live‐attenuated vaccines such as COVI‐VAC deliver a robust immune response and are associated with the long‐lasting cellular immunity.^15^

The results of a preclinical study performed to assess the immunogenicity and safety of COVI‐VAC in Syrian golden hamsters (*Mesocricetus auratus*) have demonstrated that this vaccine is safe and effective in small animal models at a single dose. Also, lower tissue viral loads and milder lung pathology were observed in Syrian golden hamsters vaccinated with COVI‐VAC compared with those inoculated with wild‐type viruses. In addition, the vaccine appeared to be resistant to reversal and could grow to a large extent.^16^ In another study, the efficiency of this vaccine was evaluated against challenge with the Beta (B.1.351) variant in the same hamsters. Twenty‐seven days' postvaccination by COVI‐VAC, animals were challenged intranasally with wild‐type SARS‐CoV‐2 Beta variant. The vaccine could prevent weight loss following the challenge with the heterologous Beta variant of SARS‐CoV‐2, B.1.351. Ultimately, it was concluded that COVI‐VAC is protective against heterologous challenge with SARS‐CoV‐2 Beta.^17^

COVI‐VAC, entered the phase 1 clinical trial in the first week of January 2021, was designed as a randomized, double‐blind, placebo‐controlled, dose‐escalation trial; 48 volunteers were tested in this trial (NCT04619628). Subjects met the following criteria: (1) men and women aged 18–30 years old, (2) negative COVID‐19 Clear test, (3) not being pregnant for women, (4) no history or current evidence of coronary heart disease, chronic obstructive lung disease, hypertension, diabetes, and other underlying diseases.^18^ In September 2021, Codagenix presented the ongoing phase 1 clinical trial of the COVI‐VAC studies at the IDWeek 2021 annual conference. The achieved data indicated that COVI‐VAC is well tolerated, with no significant adverse events reported across the 48 patients enrolled. Also, the vaccine administration resulted in minimal viral shedding, at levels lower than those likely cause the subsequent transmission of COVID‐19. According to this report, COVI‐VAC stimulates serum and mucosal antibody immune responses.^19^

NCT05233826 is an ongoing phase 1 clinical trial study evaluating the safety and immunization of COVI‐VAC as a booster dose in 30 healthy adults previously vaccinated with authorized mRNA or adenovirus vectors vaccine against COVID‐19. There is still no available report on its results.^20^ ISRCTN15779782 is an ongoing large, international, randomized controlled phase 3 clinical trial designed to provide adequate evidence of the safety and efficacy of this vaccine and is supported by the World Health Organization (WHO). The volunteers, healthy adults (aged ≥ 16 years) were randomly allocated either to placebo or vaccine group.^21^ No results from this phase have been published so far.

## 3 CoronaVac VACCINE

CoronaVac, also known as the Sinovac COVID‐19 vaccine, is a two‐dose β‐propiolactone (BPL)‐inactivated aluminum hydroxide‐adjuvanted SARS‐CoV‐2 vaccine produced by Sinovac Research and Development Co., Ltd. CoronaVac is one of the eight emergency use listing vaccines approved by WHO in 54 countries all over the world.^22^ A double‐blind, placebo‐controlled, phase 1/2 clinical trial (NCT04383574) of this vaccine was performed in 422 healthy adults aged 60 years and older in China. In phase 1, participants (*n* = 72) received a 3‐μg or 6‐μg vaccine or placebo. In phase 2, participants (*n* = 350) were given either CoronaVac at 1.5, 3, or 6 µg per dose or placebo. In the safety populations from both phases, any adverse reaction within 28 days after injection occurred in 20% of participants in the 1.5‐μg group, 20% in the 3‐μg group, 22% in the 6‐μg group, and 21% in the placebo group. All adverse reactions were mild or moderate in severity, and injection site pain was the most frequently reported event. In phase 1, seroconversion after the second dose was observed in 100% of participants in the 3‐μg group and 95.7% in the 6‐μg group. In phase 2, seroconversion was identified in 90.7% of participants in the 1.5‐μg group, 98% in the 3‐μg group, and 99% in the 6 μg group. There were no detectable antibody responses in the placebo group.^23^

To assess the immune persistence of a two‐dose schedule of CoronaVac, and the immunogenicity and safety of its third dose in healthy adults aged 18 years and older, a double‐blind, randomized, placebo‐controlled phase 2 clinical trial was conducted. There were two vaccination schedule cohorts: days 0 and 14 (cohort 1) and days 0 and 28 (cohort 2) vaccination cohorts. Half of the subjects in each cohort were selected to receive an additional dose 28 days after the second dose, and the other half of the subjects were chosen to receive the third dose 6 months after the second dose. The results of these trials showed that a two‐dose schedule of CoronaVac could generate favorable immune memory. Although neutralizing antibody titers decreased to near or below the lower limit of seropositivity 6 months after the second dose, the third dose given 8 months after the second dose was highly effective at recalling a SARS‐CoV‐2‐specific immune response.^24^

Phase 3 clinical trial was performed to evaluate the efficacy and safety of CoronaVac vaccinated healthcare workers who treated patients with COVID‐19 in Brazil. A total of 12,688 volunteers participated in the study, conducted between July 21 and December 16, 2020. All participants received at least one dose of the vaccine or placebo. Of this total, 9823 cases received both doses. According to the results, the vaccine efficacy against symptomatic COVID‐19 and hospitalization were 50.7% and 100%, respectively. Most adverse events were mild/moderate, and most of the common adverse events were pain at the injection site, headache, fatigue, and myalgia. Furthermore, there were few allergic reactions, all grade 1 or 2.^25^, ^26^ Another phase 3 clinical trial of CoronaVac was conducted in Turkey on 10,218 individuals aged 18–59 years with no history of COVID‐19 and negative results of real‐time polymerase chain reaction (PCR). Participants received the vaccine or placebo on days 0 and 14. During the 43‐day follow‐up period, nine real‐time PCR confirmed COVID‐19 cases were observed in the vaccine group and 32 cases in the placebo group 2 weeks after receiving the second dose. Vaccine efficacy was finally reported as 83%. The frequencies of adverse reactions were 18.9% in the vaccine group and 16.9% in the placebo group, with no fatalities or grade 4 side effect. Fatigue as the most frequent systemic adverse event was detected in 8.2% and 7% of the vaccine and placebo groups, respectively.^27^

The effectiveness and efficiency of CoronaVac were assessed in various investigations. Li et al. evaluated the vaccine against the Delta variant in China. Study participants were 18–59 years old, and the majority (61.3%) were vaccinated with the CoronaVac vaccine. Overall, the vaccine effectiveness for two‐dose vaccination was 59.0% against COVID‐19. The efficiency rate was 70.2% against moderate and 100% against severe COVID‐19.^13^ According to a prospective national cohort study conducted by Jara et al.^28^ on participants aged 16 years or older in Chile, the adjusted vaccine effectiveness in 10.2 million full vaccinated participants was 65.9%, 86.3% 87.5%, 90.3%, and for the prevention of COVID‐19, COVID‐19‐related death, hospitalization, and ICU admission, respectively. Ranzani et al. evaluated the efficacy of the CoronaVac in elderly people during a Gamma variant‐ associated epidemic of COVID‐19 in Brazil. The study included 43,774 adults aged ≥ 70 years who underwent reverse transcription (RT)‐PCR testing for SARS‐CoV‐2 from January 17 to April 29, 2021. Adjusted vaccine effectiveness against symptomatic COVID‐19 was 24.7% at 0–13 days and 46.8% at ≥14 days after the second dose. Also, the effectiveness against hospital admissions and deaths was 55.5% and 61.2%, respectively, at ≥14 days after the second dose.^29^ Cerqueira‐Silva investigated the influence of age on CoronaVac effectiveness and the duration of protection in 75,919,840 Brazilian cases from January 18 to July 24, 2021. Based on their results, vaccination with CoronaVac was effective against SARS‐CoV‐2 infection and highly efficient against hospitalization, ICU admission, and death in individuals up to 79 years. The vaccine efficacy against death was 67.2% among people of 80–89 years, while it was 33.6% in people above 90 years of age. Furthermore, the post‐vaccination daily prevalence rate signifies a stepwise increase from younger to elder decades of life.^30^

Phase 4 clinical trial (NCT04756830) of CoronaVac is ongoing. In this phase, the safety and immunogenicity of the vaccine are assessed against COVID‐19 in individuals over 18 years of age during 24 months of follow‐up.

## 4 VLA2001 VACCINE

Valneva, the French specialty vaccine company, designed and commercialized prophylactic vaccines for infectious diseases, such as Lyme disease, Japanese encephalitis, and the chikungunya. Using established Vero cell platform, Valneva developed a highly purified vaccine, VLA2001, against COVID‐19. VLA2001 is the only COVID‐19 inactivated vaccine candidate in clinical trials in the UK. It consists of BPL inactivated the whole virus with high spike protein density, combined with two adjuvants, alum and Dynavax's CpG 1018.^31^

Preclinical studies have suggested that combination of adjuvant‐induced high titers of neutralizing antibodies is associated with a shift in the cellular immune response toward Th1.^32^ Valneva conducted several clinical trials of VLA2001, including NCT04671017 (phase 1/2) and ISRCTN73765130 (phase 2) in UK, as well as two ongoing parallel phase 3 clinical trials, NCT04864561 (UK) and NCT04956224 (New Zealand). On December 16, 2020, VLA2001 vaccine was evaluated on 153 healthy adults (aged 18–55 years) who were vaccinated with three dose levels (low, medium, and high) of the VLA2001 vaccine, twice with 2‐week intervals.^33^ The most frequent local reactions were tenderness (58.2%) and pain (41.8%), and systemic reactions included headache (46%) and fatigue (39.2%). The vaccine was exhibited to be safe and well‐tolerated and produced both humoral and cellular immune responses, with a clear dose‐dependent effect.^34^ Later, Valneva participated in the Cov‐Boost clinical trial (ISRCTN73765130) to examine seven different COVID‐19 booster vaccines. The results of this trial revealed that all studied vaccines, including VAL2001, boosted antibody and neutralizing responses after ChAd/ChAd or BNT/BNT initial course, with no safety concerns.^35^ Considering these data, the company decided to commence a phase 3 clinical trial by the end of April 2021. In phase 3 immunogenicity trial, Cov‐Compare (NCT04864561), the safety and efficacy of VLA2001 were compared with AstraZeneca's conditionally approved vaccine recruited in the UK on 4000 adult volunteers aged 18 years and older. According to the data reported by the company, VLA2001 demonstrated superiority over AZD1222 (ChAdOx1‐S) in terms of geometric mean titer for neutralization antibodies and also non‐inferiority in terms of seroconversion rates. Likewise, T‐cell responses analyzed in a subset of participants showed that VLA2001 induced broad antigen‐specific interferon (IFN)‐γ‐producing T cells reactive to the proteins S (74.3%), N (45.9%), and M (20.3%). The vaccine also demonstrated a significantly greater tolerability profile than the ChAdOx1‐S vaccine. Participants reported fewer injection site reactions (73.2% VLA2001 vs. 91.1% AZD1222) and systemic reactions (70.2% VLA2001 vs. 91.1% AZD1222).^36^ In parallel to the phase 3 trial conducted in the UK, the second phase 3 trial, VLA2001‐304 (NCT04956224) was initiated in New Zealand and included two cohorts. The open‐label cohort 1 was conducted to assess the safety and immunogenicity of two doses of the vaccine (at 28‐day intervals) in 300 subjects aged 56 years or above. The cohort 2 was carried out with nearly 600 vaccinated subjects aged 12 years or above to evaluate the immunogenicity of VLA2001 against VLA2101, another COVID‐19 vaccine candidate targeting variant strain.^37^ At the end of December 2021, Valneva announced positive homologous booster results. An excellent immune response was also observed after the third dose of VLA2001 administered 7–8 months following the second dose of primary vaccination.

The efficacy of the VLA2001 against Delta and Omicron variants was evaluated by preparing S‐protein‐expressing Pseudovirus from the ancestral SARS‐CoV‐2 virus, Delta variant, or Omicron variant. Furthermore, the serum of 30 people who participated in phase 1 and 2 trials was used to analyze the neutralization of the ancestral SARS‐CoV‐2 virus, as well as Delta and Omicron variants. All samples showed neutralized antibodies against the ancestral virus. The incidence of neutralizing antibodies against Omicron was 87%.^38^ In March 2022, the National Health Regulatory Authority of the Kingdom of Bahrain granted emergency use authorization for VLA2001.^39^

## 5 ERUCOV‐VAC VACCINE

ERUCOV‐VAC or TURKOVAC is a COVID‐19 vaccine candidate developed by the collaboration of the Turkish company Kocak Farma with the Health Institutes of Turkey. For the production of ERUCOV‐VAC, Vero E6 cells were cultivated in a multitray cell factory system. The safety and efficacy of well‐characterized Vero cell‐based inactivated vaccines make it an appealing platform for fast vaccine development and COVID‐19 implementation.^40^ Phase 1 trial (NCT04691947) of the vaccine was initiated in November 2020 with 44 participants in Turkey.^41^ On February10, 2021, ERUCOV‐VAC vaccine was evaluated on at least 250 human volunteers aged 18–64 years, which resulted in acceptable immunogenicity and safety in the phase 2 clinical trial.^42^ Overall, phase 1 and 2 trials showed the safety and immune response of the vaccine.^43^ In Turkey, Pavel and associates^40^ evaluated the preclinical immunogenicity, protective efficacy, and safety of ERUCOV‐VAC prepared in aluminum hydroxide, the most extensively used vaccine adjuvant, in three animal models, comprising BALB/c mice, transgenic mice (K18‐hACE2), and ferrets. The hCoV‐19/Turkey/ERAGEM‐001/2020 strain was employed to test the safety of ERUCOV‐VAC. In BALB/c mice, the vaccine was found to be highly immunogenic and induced a significant immune response. In K18‐hACE2 mice, the ERUCOV‐VAC vaccine displayed 100% protection against a lethal SARS‐CoV‐2 challenge. In the ferret models, viral clearance rates were similar to the safety assessment of the vaccine in upper respiratory tracts. The most prevalent minor adverse effect was discomfort at the place of the injection.^44^ ERUCOV‐VAC is presently in phase 3 clinical trial (NCT04942405). Its name was shifted to TURKOVAC in this phase.^40^

## 6 COVIran BAREKAT VACCINE

COVIran Barekat is a COVID‐19 vaccine candidate developed by Shifa Pharmed Industrial Group Company, a subsidiary of Barkat Pharmaceutical Group. SARS‐CoV‐2 virus culture on Vero monolayers cells and then virus particles were inactivated using PBL. Alum adjuvant was utilized in the formulation of COVIran Barekat to achieve an effective and robust immune response.^45^ In phase 1 clinical trial (IRCT20201202049567N1), the vaccine was evaluated on 56 healthy volunteers with the age range of 18–55 years, which led to satisfactory safety and immune response. In the second phase 1 clinical trial (IRCT20201202049567N2), the evaluation of the vaccine was started in a smaller population, that is, 32 healthy adults aged 51–75 years.^46^ Following the promising results of the phase 1, phase 2/3 clinical trial (IRCT20201202049567N3) was performed as randomized, double‐blind, parallel arms, placebo‐controlled trial. In this phase, 280 volunteers (aged 18–75 years) enrolled and were vaccinated with 5.0 mg of COVIran Barekat twice at 28‐day intervals, which resulted in favorable immune response. Phase 3 clinical trial was initiated in six cities with 2000 volunteers who were vaccinated with 5.0 mg of the vaccine two times at intervals of 28 days, which resulted in favorable efficacy in preventing mild, moderate, and severe diseases.^46^ In the evaluation of post‐vaccination signs and symptoms among Iranian health professionals who were vaccinated with COVIran Barekat vaccine, pain, tenderness, and itching were observed.^47^

## 7 BBV152 VACCINE

Covaxin (codenamed BBV152), India's first COVID‐19 vaccine, was developed by Bharat Biotech Limited in collaboration with the Indian Council of Medical Research and National Institute of Virology (NIV). The vaccine was manufactured in (Bio‐Safety Level 3) high containment facility and designed based on inactivating the whole‐virion SARS‐CoV‐2 strain NIV‐2020–770.^48^

In an animal trial on rhesus macaques, the protective efficacy and immunogenicity of BBV152 as an inactivated SARS‐CoV‐2 vaccine were assessed. The study included 20 macaques that were categorized into four equal groups. Placebo was administered to one of the groups, while the remaining groups were vaccinated with three different vaccine candidates of BBV152 at days 0 and 14. Two weeks after the second dose, all animals were challenged with SARS‐CoV‐2. From the third week after immunization, the protective response was induced with elevating SARS‐CoV‐2‐specific IgG and neutralizing antibody titers. In the vaccinated group, 7 days after infection, viral clearance was observed from bronchoalveolar lavage fluid, nasal and throat swabs, and lung tissues. Unlike the placebo group, the vaccinated groups indicated no symptom of pneumonia.^49^ BBV152 showed higher immune response and safety in phase 2 than the phase 1 trial.^50^

Ella et al. performed an investigation on 16,973 subjects with 2‐week follow‐up after the second vaccination and reported 130 cases of symptomatic COVID‐19, 24 vaccine and 106 placebo participants. The overall efficacy of the BBV152 vaccine was found to be 77.8%. One case in the vaccine group and 15 cases in the placebo group experienced severe symptomatic COVID‐19, suggesting a vaccine efficacy of 93.4%. However, the efficacy against asymptomatic COVID‐19 was 63.6%. BBV152 showed 65.2% protection against the SARS‐CoV‐2 variant of concern, B.1.617.2 (Delta). There was no report of anaphylaxis or deaths in relation to the vaccine. BBV152 was well‐tolerated with an incidence of AVs over a median of 146 days, which was less than that detected in other COVID‐19 vaccines.^51^ The safety and immunogenicity of BBV152, adjuvanted with aluminum hydroxide gel (Algel) or a novel TLR7/8 agonist chemisorbed Algel, were examined in another study. In this regard, a strain of SAS‐CoV‐2 and a Vero cell platform were selected to induce a highly purified inactivated antigen, BBV152. Immunogenicity was evaluated at two (3 and 6 µg) concentrations and with two varied adjuvants in three animals, including mice, rats, and rabbits. The vaccine produced significantly high antigen‐binding and neutralizing antibody titers, at both antigen concentrations and in all animals, with superb safety profiles. The inactivated vaccine, comprising of TLR7/8 agonist adjuvant‐induced Th1, biased antibody responses with increased IgG2a/IgG1 ratio and elevated levels of SARS‐CoV‐2‐specific IFN‐γ + CD4 T lymphocyte response. These findings confirm further development of phase 1/2 clinical trial, particularly in humans.^52^ BBV152 was reported to be the first inactivated SARS‐CoV‐2 vaccine that induced a Th1‐biased response with few significant side effects.^53^ The most prevalent adverse effects of the BBV152 vaccine among Birjand healthcare professionals were injection site pain, muscular soreness, lethargy, fever, and headache. Also, the most relevant factors in the prevalence of adverse effects from vaccination were age and gender.^54^

During the phase 3 trial, which included 25,800 patients aged 18–98 years (2433 above the age of 60 and 4500 with comorbidities), vaccination with BBV152 demonstrated an temporary efficacy of 81% in preventing COVID‐19.^55^

## 8 QazCovid‐in VACCINE

QazCovid‐in, commercially known as QazVac vaccine, was developed by the Research Institute for Biological Safety Problems in Kazakhstan. It is a two‐dose, intramuscular, and formalin‐inactivated vaccine adjuvanted with aluminum hydroxide.^56^ Phase 1/2 clinical trial (NCT04530357) was conducted in two parts to evaluate the safety, tolerability, and immunogenicity of vaccine based on the comparison of antibody titers in serum samples before and after vaccination, which was measured by microneutralization test (MNA) and enzyme linked immunosorbent assay (ELISA) tests. QazVac vaccine testing on 44 subjects aged 18–50 years demonstrated its favorable tolerability in phase 1 part of phase 1/2 clinical trial, and MNA showed seroconversion in 100% of subjects after two doses of the vaccine.^57^

Phase 2 clinical trial was performed on 200 healthy adults (aged 18–49 or ≥50 years) who were randomized into four equal‐sized groups based on single (day 1) or double (day 1 and 21) vaccination protocol, to assess the immunogenicity of the vaccine. On day 21 of the vaccination, the number of seroconversions reached 92% in the one‐dose group and 94% in the two‐dose group. Observation of adverse events within 7 days after the first or the second vaccinations presented mild local and systemic reactions, which diminished significantly in the second dose.^57^

Following promising results in phase 2, the final phase 3 clinical trial (NCT04691908) was carried out on 3000 volunteers aged 18 years and older in three clinical centers with a 180‐day follow‐up period of monitoring to evaluate the QazCovid‐in vaccine preventive efficacy against COVID‐19. Appropriate subjects were randomly divided into placebo and two‐dose vaccine (days 1 and 21) groups. Induction of humoral immunity in two groups was assessed by MNA and ELISA and cellular immune responses by measuring the cytokine levels of IFN‐γ, interleukin (IL)‐6, and tumor necrosis factor (TNF)‐α. Results disclosed that the administration of two doses of the vaccine induced a fourfold increase in MNA titers in the majority (99%) of individuals on day 42. Furthermore, significant elevation was observed in the levels of IFN‐α, IFN‐γ, IL‐6, and TNF‐α in the vaccine group on days 90 and 180 compared to day 1 in both vaccine and placebo groups, a remark for cellular immunity induction. The preventive efficacy of vaccination was defined as the ratio of RT‐PCR‐confirmed COVID‐19 cases of any severity since day 14 after the first dose administration or later in the vaccine group compared to placebo group. As a result, vaccine efficacy amounted to 82.0%.^58^

In a study carried out from December 2020 to July 2021, 45 confirmed positive virus samples were sequenced to evaluate the immune protection of the vaccine against different variants of SARS‐CoV‐2, such as Wuhan Hu‐1‐like viruses, as well as Alpha and Delta new variants. Based on the results, the three variants were circulating almost equally, indicating the efficacy of the vaccine against new presented variants. For this reason, it was hoped that QazCovid‐in vaccine could be preventive against the new variant of SARS‐CoV‐2, Omicron; however, there are no relevant data.^58^

## 9 VERO CELLS VACCINE

This vaccine is a two‐dose inactivated SARS‐CoV‐2 vaccine (Vero cell) adjuvanted with aluminum hydroxide and produced by the Institute of Medical Biology and the Chinese Academy of Medical Sciences. A preclinical trial study of Vero cell vaccine was executed on rodent Sprague Dawley rats after multiple intramuscular injections. Examination of weight and food intake, eye and urine routine, hematologic properties, serum biochemistry, neutralizing antibody, determination of CD4^+^ T cell and CD8^+^ T cell, and pathological analysis were indices investigated in experimental and negative control groups. After 14 days since the last administration, the neutralizing antibodies in the low‐ and high‐dose vaccine groups began to appear. There were no regular alterations or notable stimulating reactions related to the vaccine injection in rats.^59^

SARS‐CoV‐2 vaccine (Vero cells) phase 1 clinical trial (CTR20200943 and NCT04412538) was conducted in May 2020. In this trial, 192 eligible adults (aged 18–59 years old) were selected and distributed to two groups with varied immunization schedules on days 0 and 14 or days 0 and 28; each group received a placebo and three vaccine doses (50,100, and 150 EU). At days 14 and 28 after vaccine administration, the seroconversion rates were 87.5% and 79.2% (50 EU), 100% and 95.8% (100 EU), and 95.8% and 87.5% (150 EU). Serum cytokine levels of IL‐6, IL‐1, IL‐2, TNF‐α, and IFN‐γ in the three‐dose groups were parallel with the placebo group. In addition, no significant differences were observed between the two groups regarding various T‐cell populations counts in the peripheral blood. Mild pain and redness at the injection site and slight fatigue were the most usual adverse events. There were no severe side effects or serious reactions within 28 days. Therefore, vaccine was considered safe and without immunopathologic impacts.^60^

Vero cell vaccine still has not been approved for use. Phase 2 and 3 clinical trials are currently recruiting or have not yet started. This is why we could not find published papers or data relevant to vaccine efficacy against different SARS‐CoV‐2 variants and probable adverse effects.^61^, ^62^

## 10 BBIBP‐CorV VACCINE

The BBIBP‐CorV, also known as the Sinopharm COVID‐19 vaccine, was manufactured by Sinopharm's Beijing Institute of Biological Products (BBIBP). The vaccine was developed from two inactivated vaccines (WIV04 and HB02) that were based on two different SARS‐CoV‐2 isolates from patients in China.^2^ HB02 strain is now recognized as BBIBP‐CorV. Both vaccines are made of virus particles cultured in the Vero cell line to obtain the spike protein that has lost the ability to cause disease followed by BLP inactivation, and adjuvanted with aluminum hydroxide.^63^ Both vaccine candidates passed the phase 3 clinical trials, and the WHO ultimately approved Chinese COVID‐19 vaccine for emergency use in many countries and regions on May 7, 2021.^64^

According to a preclinical study by Wang et al.,^2^ vaccination with BBIBP‐CorV could generate high levels of neutralizing antibody titers in mice, rats, guinea pigs, rabbits, and non‐human primates to help protection against SARS‐CoV‐2. In a randomized, double‐blind, placebo‐controlled phase 1/2 trial conducted in China, 192 subjects participated in phase 1 and randomly received the vaccine at the doses of 2, 4, or 8 μg (for age groups 18–59 years and 60 years) or placebo. In phase 2, 448 individuals were enrolled and randomly given the vaccine or a placebo. The findings of these trials demonstrated that BBIBP‐CorV is safe and well‐tolerated at all doses tested in two age groups. Two 4‐μg doses of the vaccine on days 0 and 21 or days 0 and 28 resulted in higher neutralizing antibody titers than a single 8‐μg dose or 4‐μg dose on days 0 and 14. Moreover, the two‐dose vaccine administration (at doses 2, 4, and 8 μg) on days 0 and 28 in both age groups induced seroconversion in 100% of subjects.^65^ Al Kaabi et al. published an interim analysis of phase 3 randomized, double‐blind trial in adults aged 18 years and older with unknown history of COVID‐19 in the United Arab Emirates (UAE) and Bahrain to evaluate two SARS‐CoV‐2 strains, WIV04 and HB02. Participants were randomized to receive one of following injections: WIV04 (5 μg/dose; *n* = 13,459), HB02 (4 μg/dose; *n* = 13,465) or aluminum hydroxide (*n* = 13,458) as the control group. The injections were given intramuscularly 21 days apart. Trial findings indicated that the vaccine efficacy was 72.8% (95% CI: 58–82) for WIV04 and 78.1% (95% CI: 65–86) for HB02, which significantly reduced the risk of symptomatic COVID‐19. Serious adverse events were uncommon in the three groups.^66^ Wang et al. created recombinant SARS‐CoV‐2 Pseudoviruses, including the wild‐type spike protein, the D614G mutation, B.1.1.7 (Alpha), and B.1.351 (Beta) variants to measure the resistance of SARS‐CoV‐2 variants to neutralization elicited by infection or vaccination. They obtained serum from 50 participants 2–3 weeks after receiving the second dose of inactivated virus vaccines (BBIBP‐CorV or CoronaVac). Furthermore, convalescent serum was obtained from 34 patients 5 months after infection with COVID‐19. Although the geometric mean titers (GMTs) of neutralization against the variations were not substantially different from the GMTs against the wild‐type virus in the 25 BBIBP‐CorV vaccine serum samples, 20 samples demonstrated total or partial loss of neutralization against B.1.351. Also, the B.1.1.7 variation showed minimal resistance to the neutralizing activity of convalescent or vaccine serum. Altogether, the B.1.351 variant exhibited higher convalescent and inactivated vaccine serum neutralization resistance than the wild‐type virus.^67^ Vályi‐Nagy et al. made a comparison between the humoral and cellular immune responses elicited by the SARS‐CoV‐2 vaccines, including BBIBP‐CorV and BNT162b2 (mRNA‐based). According to their findings, the BBIBP‐CorV vaccine produced anti‐receptor binding domain IgG and anti‐spike protein IgG and IgA antibodies in healthy people, which were much lower than after BNT162b2 vaccination but higher than in convalescent patients. However, the total number of IFN‐γ‐secreting, virus‐specific T cells, differed little between mRNA vaccine and inactivated virus vaccinated participants. They concluded that two doses of the BBIBP‐CorV vaccine could induce the modest anti‐SARS‐CoV‐2 antibody and robust T‐cell responses in healthy adults.^68^

In a recent survey, Mira Mousa et al. assessed the effectiveness of the inactivated vaccine BBIBP‐CorV (Sinopharm) and the mRNA vaccine BNT162b2 (Pfizer‐BioNTech) on the possibility of hospitalization in COVID‐19 confirmed patients who were immunized with one of two vaccine(s) or unvaccinated during Delta (B.1.617.2) variant outbreak in UAE. Their results reflected that the admission rate in two‐dose vaccinated people were much lower than unvaccinated individuals (BBIBP‐CorV: 6.3%; BNT162b2: 1.2%; unvaccinated: 24.1%), and vaccines efficacy calculated as 95% and 98%, respectively (Table 1). ^69^ In another recent survey, Jingwen Ai et al. investigated the efficacy of heterologous (BBIBPCorV) and homologous (BBIBPCorV/ZF2001) booster vaccines against the prototype virus, as well as Beta, Delta, and Omicron variants. Their results indicated that 14 days after the third vaccination, seropositivity against Omicron occurred in 100% of samples, but neutralization titers against Omicron reduced by 11.65‐fold and 7.94‐fold, respectively, compared with prototype and Delta variants, which are likely related to mutations in the spike protein.^70^

**TABLE 1:** Characteristics of live‐attenuated and inactivated COVID‐19 vaccines

Vaccine name	Developer	Route of administration/ dose	Clinical stage	Type of subunit and structure	Type of adjuvant	Efficacy	Side effects	Reference
COVI‐VAC	Codagenix/Serum Institute of India	IN/1	Phase 3	Attenuated vaccine through codon deoptimization techniques	No Adjuvant	COVI‐VAC stimulates serum and mucosal antibody immune responses based on phase 1 trial	Well‐tolerated, with no significant adverse events reported across the 48 patients enrolled in phase 1	16, 19
Corona VAC	Sinovac Biotech	IM/2	Phase 4	Whole‐virion vaccine inactivated with BPL	Aluminum hydroxide	50.7% against symptomatic COVID−19 and 100% against hospitalization based on phase3 trial in Brazil, 65.9% based on study in Chile and 83% based on phase 3 in Turkey	Mild/moderate, and most of the common adverse events were pain at the injection site, headache, fatigue, and myalgia	26, 27, 28
VLA2001	French biotechnology company Valneva SE	IM/2	Phase 3	Vero cell‐based inactivated vaccines	Alum and CpG 1018	Good humoral and cellular immune responses, based on phase 1/2 trial. Superiority against ChAdOx1‐S in terms of geometric mean titer for neutralization antibodies based on phase 3.	Safe and well‐tolerated tenderness, pain, headache and fatigue	34, 35, 36
TURKOVAC	Turkish Kocak Farma	IM	Phase 3	Vero cell‐based inactivated vaccines	Aluminum hydroxide	Well immunogenicity (based on phase 2 in the NJ) and 100% (based on Pavel et al. in Turkey)	Discomfort at the place of the injection	40, 84
COVIran Barekat	Shifa Pharmed Industrial Group	IM	Phase 3	Vero cell‐based inactivated vaccines	Alum adjuvant	Well efficacy (based on phase 3 in the Iran)	Pain, tenderness and itching	45, 47, 85
Covaxin	Bharat Biotech Limited	IM	Phase 3	Inactivating the whole‐virion SARS‐CoV−2 strain NIV−2020–770	Algel‐IMDG	77.8% (based on Ella et al. in India) and 81% interim efficacy (based on Phase 3 in India)	Injection site discomfort, muscular soreness, lethargy, fever, and headache	48, 51, 53, 54
QazCovid‐in	Science Committee of the Ministry of Education and Science of the Republic of Kazakhstan	IM /2	Phase 4	Whole‐virion formaldehyde‐inactivated	Aluminum hydroxide	82·0% (based on confirmed positive vaccinated subjects by RT‐PCR results on phase 3 in Republic of Kazakhstan)	Commonly mild (Pain at the injection site, Swelling, Hyperemia, Fever, Headache and Weakness on 7 days after both vaccinations based on phase 3)	57, 58
SARS‐CoV−2 vaccine (Vero Cell)	Institute of Medical Biology and the Chinese Academy of Medical Sciences	IM/2	Phase 3	Whole‐virion formaldehyde‐inactivated	Aluminum hydroxide	Variable, depended on vaccine dose and spent time after administration. 100% for 50 EU on day14 based on phase I data	Mild pain and redness at the injection site and slight fatigue were the most usual adverse events	^60^
Sinopharm (BBIBP‐CorV) vaccine	Sinopharm's Beijing Institute of Biological Products (BBIBP)	IM/2	Phase 4	Whole‐virion formaldehyde‐inactivated (WIV04 and HB02 strains)	Aluminum hydroxide	78.1%, based on phase 3 clinical trial in UAE	Adverse reactions 7 days after each injection occurred in 41.7%–46.5% of subjects. Serious adverse events were uncommon	2, 66

Abbreviations: IM, Intramuscular; IN, Intranasal; NR, Not Reported.

## 11 COMPARISON OF INACTIVATED PLATFORM WITH OTHER PLATFORMS

So far, different types of vaccines have been designed to prevent COVID‐19 and have been tested in various laboratory phases. In general, these vaccines fall into four categories: nucleic acid, viral vector, whole pathogen, and protein vaccines.^71^ Vaccines based on nucleic acids use genetic materials (mRNA, DNA, and siRNA) that can produce specific proteins inducing antibodies and stimulate memory T cell.^72^ Viral vector vaccines are genetically modified and safe viruses that fail to produce specific immunogenic proteins. Protein‐based vaccines are actually whole recombinant spike proteins packaged in nanoparticles.^71^ Inactivated vaccines are a type of whole pathogen vaccine. This type of vaccines are the oldest and most well‐known vaccines because they contain the whole attenuated or inactivated pathogen that produces the immune response.^73^ Each vaccine has its own advantages and disadvantages that distinguish it from another.^74^ Ease of fabrication, cheapness, strong immune response, and immune stimulation by T and B cells are significant features of whole microbial vaccines. In addition, whole microbial vaccines provide faster immunity than vaccines based on nucleic acid, protein, and viral vector. The remarkable point is that nucleic acid‐based vaccines are slower to develop than other types of vaccines, but they are safe and effective.^71^

## 12 INACTIVATED VACCINES IN PATIENTS WITH NEW VARIANTS

One of the most important issues in relation to vaccines is the emergence of new strains of SARS‐CoV‐2. Natural selection is responsible for choosing mutations to maintain the survival, proliferation, and fitness of organisms.^75^ Therefore, the immunogenicity of vaccines against new strains requires constant evaluation. Research has emphasized that the Omicron variant declines the neutralization ability of vaccines by evading neutralizing antibodies, which reduces immune responses. Decreased immune responses were found to be even less than in Mu and Beta strains.^76^ Studies have pointed out that after injecting two doses of inactivated vaccine, a booster is needed. Booster vaccines can be a variety of mRNAs, protein subunits, and inactivated vaccines.^70^, ^77^, ^78^ Immunoglobulin immune responses to Omicron variant occur only after booster injection.^79^

In one study, the protective effect of BBIBP‐CorV against new variants, B.1.1.7 in the UK (Alpha) and B.1.351 in South Africa (Beta), and Wuhan‐1 reference strain (wild‐type) was assessed. The results showed that BBIBP‐CorV was more effective against B.1.1.7 than B.1.351.^80^ The efficacy of BBV152/COVAXIN against the Alpha strain and COVI‐VAC vaccine against Beta strain has also been highlighted.^17^, ^81^ The results of an investigation on the protective effect of the CoronaVac against seven variants of SARS‐CoV‐2 portrayed that the efficacy of the wild‐type was similar to the D614G, B.1.1.7, and B.1.429 variants. Neutralization property in the B.1.526, P.1, and B1.351 was explored to be mitigated severely.^82^ COVI‐VAC vaccine produces neutralizing antibodies against both Omicron and Delta variants.^83^ Little is known about the effectiveness of other inactivated vaccines on new variants, and scientists have to continually monitor the performance of vaccines.

## 13 CONCLUSION

Producing effective vaccines on the basis of inactivated whole virion against SARS‐CoV‐2 infection as a conventional technology, is a reliable option. Herein, we reviewed nine vaccines, which are passing phase 3 and 4 clinical trials, from different countries, including France, China, India, Iran, Turkey, and Kazakhstan. In these vaccines, a wild‐type virus particle commonly cultured in Vero cell line is inactivated by BPL or formaldehyde, adjuvanted with substances such as aluminum hydroxide, CpG 1018, or each of them in VLA2001. The occurrence of adverse events in all reviewed inactivated vaccines was uncommon, and there were no reports on anaphylaxis or vaccine‐related deaths. In Iran, COVIran Barekat, Covaxin (Bharat Biotech), and BBIBP‐CorV (Sinopharm) are three inactivated COVID‐19 vaccines approved for use. Overall, two or three intramuscular doses of these vaccines can induce sufficient cellular responses, a fault attributed to inactivated vaccines. Taken together, despite all attempts to produce effective vaccines, the appearance of new mutated SARS‐CoV‐2 strains is a critical challenge. The efficiency of available vaccines against new strains is a subject that needs further investigation.

## CONFLICT OF INTERESTS

The authors declare that they have no conflict of interest.

## AUTHOR CONTRIBUTIONS

SK, MA, SA, MK, HG, NS, and MH contributed to comprehensive research and wrote the paper. AS participated in editing the manuscript. Notably, all authors have read and approved the manuscript.

## Data Availability

The authors confirm that the data supporting the findings of this study are available within the article.
